# Determinants of severe acute malnutrition among children less than five years visiting health centers in Leqa Dulacha District, East Wallaga Zone, Oromia Region, Ethiopia: A case control study

**DOI:** 10.1002/hsr2.1939

**Published:** 2024-05-02

**Authors:** Garoma Begna, Haile Bikila, Bayise Biru, Debelo Diriba, Chimdesa Tolera, Ra'el Dessalegn, Temesgen Tafesse, Dessalegn Amenu

**Affiliations:** ^1^ Department of Public Health Emergency Management Leqa Dulacha District Health Office, East Wallaga Zone Nekemte Ethiopia; ^2^ Department of Public Health, Institutes of Health Sciences Wallaga University Nekemte Ethiopia; ^3^ Department of Health and Nutrition Food for Hungry Ethiopia, East Wallaga Zone Nekemte Ethiopia; ^4^ Department of Public Health Management Leqa Dulacha District Health Office, East Wallaga Zone Nekemte Ethiopia; ^5^ Department of Health Science Sibu Sire District, Sibu Sire Health Center, East Wollega Zone Health Nekemte Ethiopia; ^6^ Armauer Hansen Research Institute Addis Ababa Ethiopia; ^7^ Department of Biology, College of Natural and Computational Science Wollega University Nakemte Ethiopia

**Keywords:** children aged 6–59 months, determinants, Ethiopia, severe acute malnutrition

## Abstract

**Background:**

In underdeveloped nations like Ethiopia, severe acute malnutrition (SAM) is one of the most pressing public health issues. Despite efforts to pinpoint the causes of SAM, the impact of parents' drug usage on their children's nutritional status remains unclear and unresolved.

**Objective:**

The purpose of this research was to determine the risk factors for SAM in children under five who were attending medical facilities in the Leqa Dulacha district.

**Materials and Methods:**

A health facility‐based case‐control study was carried out from March 1 to July 30, 2022, with 256 children under the age of five. Random sampling was used to identify study participants in a methodical manner. Mothers and other child caretakers were interviewed using a structured questionnaire and anthropometric measurements were performed using standardized, calibrated equipment. Epi‐data version 3.1 was used to code and enter the data, and it was then exported to IBM SPSS for analysis. An analysis of multivariable binary logistic regression was conducted, and the measure of association employed was the adjusted odds ratio (AOR), with a 95% confidence interval (CI).

**Results:**

A total of 96.5% of respondents responded. SAM in children was significantly correlated with the following factors: parent alcohol consumption [AOR = 3.142; 95% CI = (1.104, 8.945)]; child illness in the previous 15 days [AOR = 4.122; 95% CI = (1.686, 10.07)]; poor dietary diversity [AOR = 3.044; 95% CI = (1.189, 7.788)]; household food insecurity [AOR = 4.024; 95% CI = (1.544, 10.490)]; and parent chewing chat [AOR = 3.484; 95% CI = (1.329, 9.134)].

**Conclusions:**

A number of factors have been linked to SAM in children, including the use of health services, the child's illness within the previous 15 days, food security, child feeding practices, and parent substance use. Therefore, it is important to emphasize the value of health education programs on child feeding habits, particularly the significance of dietary diversity, and to work together to modify the way that parents raise their children.

## 1 BACKGROUND

The most severe type of undernutrition is known as severe acute malnutrition (SAM), which is characterized by a significant loss of muscle mass and weight (wasting), which results in the development of very low weight for height and length.^1^ There are several ways that SAM in children can appear; among the later signs in children with kwashiorkor are changes in skin pigmentation, peeling and cracking, and changes in hair texture, thinness, and discoloration.^2^, ^3^ Furthermore, the disease affects the nutritional status of a child by either increasing nutrient and energy requirements or reducing the child's appetite and body's utilization to ingested food.^4^

A number of fundamental problems, including poverty, political instability, natural disasters, climate change, and a lack of policy initiatives, are variables that contribute to the underlying causes of SAM.^5^, ^6^ Approximately three million children die each year worldwide, and of those fatalities, 45% are caused by conditions that are either directly related to or worsened by SAM.^6^ SAM is directly or indirectly responsible for 50% of under‐five mortality in Ethiopia.^7^ One of the top three causes of death in Ethiopia is SAM, with an estimated 8.5% cause‐specific mortality rate associated with it.^8^

Global literature indicates that poverty, parental illiteracy, poor feeding practices, large family sizes, nonexclusive breastfeeding, diarrhea, low birth weight, immunization status, disturbed families, mother hand washing habits, and repeated pregnancies contribute to acute malnutrition in children aged 6–59 months.^5^, ^9^, ^10^, ^11^, ^12^, ^13^, ^14^, ^15^, ^16^, ^17^, ^18^ Ethiopia's acute malnutrition remains a significant issue, but its causes are poorly understood and inconsistent across studies. Previous research lacks evidence across all regions and uses cross‐sectional designs, making it difficult to identify factors affecting under five children. Understanding these determinants at different levels in society is crucial, as they are hierarchically interrelated. Hence, to investigate the effects of parent's life style on children's nutritional status, it needs to conduct further studies. Therefore, this study was conducted to identify determinants of SAM among under‐5‐year‐old children visiting health centers in Leqa Dulacha district, East Wollega Zone, Ethiopia.

## 2 METHODS AND MATERIALS

### 2.1 Study design, area, and period

The study was conducted in East Wollega Zone, Leqa Dulecha District and Leqa Delecha, Leqa Dulacha Health center, East wollega Zone, Oromia Regional state, Western Ethiopia from March 1, 2022 to July 30, 2022. East Wollega zone is one of Oromia national regional state's 18 administrative zones, located west of Addis Abeba. It covers 14,255 square kilometers and has a population of 1,531,380 (one million five hundred thirty‐one thousand three hundred eighty) according to the 2007/2008 census. The people's economy is centered on subsistence farming and livestock keeping. Currently, the zone has two government‐owned hospitals and 58 health facilities. A health facility based case control study was conducted from March 1, 2022 to July 30, 2022.

### 2.2 Source and study population

Source population was all children of under 5 years old with mother's/caregivers pair visiting the four health centers in the Leqa Dulacha district for different health care services. Study population was all 6–59 months old children with SAM who visited health facilities during data collection periods. Anthropometric measurement indices: weight‐for‐height/length (WFH/L) <−3 or mid‐upper arm circumference (MUAC) <11.5 cm and or presence of bilateral oedema related to malnutrition was used to identify cases.

### 2.3 Inclusion criteria

Children aged 6–59 months who attended or were hospitalized to hospitals with acute malnutrition (MUAC 12.5 cm or with bilateral pitting edema of nutrition origin) and their caretakers/mothers who gave informed consent were selected as cases. Children aged 6–59 months who attended hospitals (MUAC 12.5, without bilateral pitting edema of nutritional cause) with their mothers/caretakers who gave informed consent were included as controls.

### 2.4 Exclusion criteria

**Figure 1:**
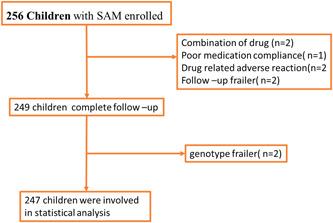
Inclusion and exclusion eligibility criteria, flow chart. SAM, severe acute malnutrition.

Children who had physical deformities (children born without hands due to congenital deformities, wounded, and burned hands) which make anthropometric measurements inconvenient were excluded from the study is described in below flow chart (Figure 1).

### 2.5 Selection of cases

Children aged 6–59 months hospitalized during the data collecting period were assigned to one of two hospitals based on the previous month's critically malnourished child flow. Children with moderate to severe malnutrition and a MUAC of 12.5 cm or bilateral pitting edema of nutritional etiology were included in the study.

### 2.6 Controls selection

Children without malnutrition (MUAC of 12.5 cm) and without bilateral pitting edema of nutritional etiology were chosen as controls from the same hospital as cases.

### 2.7 Sample size determination

**Figure 2:**
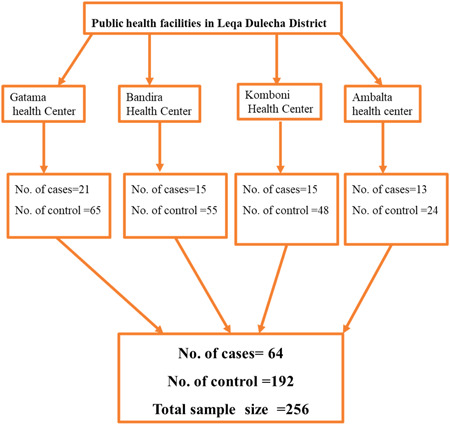
Schematic presentation of the sampling procedure of the study, 2022.

Sample size has been calculated by double population proportion formula by using Epinfo version: 7.2.4.0. Different sample size has been calculated by considering for different determinants of SAM from previous studies (27, 32, 42). Low level maternal decision autonomy in the household was considered as a major determinant factor of SAM because it gives maximum sample size relative to the other determinant factors. The assumptions used for calculation are detecting 2.48 times higher odds of low level maternal decision autonomy in the household among the cases than among controls; 41.8% of controls and 64.1% of cases will respond that maternal decision autonomy in the household is low. With a 95% confidence level, 80% power, using 3:1 controls/case ratio, adjusting for assumed 10% nonresponse rate, the total sample size was 256 (64 cases and 192 controls) (Figure 2).

### 2.8 Sampling techniques and procedure

Laka Dulach district has 4 health centers (Gatama health Center, Bandira Health Center, St Daniel Komboni Health Center, and Ambalta health center). Study participants were selected from all the four health centers. The numbers of cases to be selected from each health center were determined depending on the proportion of cases of SAM of children with under 5 years' old reported to the district health office from each health center in the preceding year. The numbers of controls to be selected from each health center were determined depending on the number of cases assigned to be selected from the same health center. Both cases and controls were selected by systematic random sampling techniques.

### 2.9 Data collection tools

Based on their past expertise collecting data, five diploma nurses and two supervisors were chosen for the data collection team, as well as data collectors' daily operations. Anthropometric measurements were used to establish the nutritional condition of the children, and standardized interviewer‐administered questionnaires were used to gather data from mothers and other child caregivers. After examining several relevant literatures, data collection tools were created to meet the study's aims. For fieldwork purposes, the questionnaire was developed in English and translated into the local language, afaan oromo. Language experts then checked the consistency of the translation by translating it back into English. The following areas comprised the questionnaire: MCH service utilization, dietary considerations, child features and diseases, sociodemographic characteristics, child caring feeding practices. Mothers were asked to validate and check their experience related to alcohol consumption, smoking cigarettes, and chewing chat habits. In addition, all related data was adapted from the World Health Organization instrument for stepwise surveillance for child malnutrition and by reviewing different literatures which were related to determinants of SAM. For the time being Household Food Insecurity Access Scale (HFIAS) questionaries' were not include in this study.

### 2.10 Data quality assurance

To ensure the quality of data to be collected, training was given for both data collectors and supervisors for 2 days before starting data collection. During training, standardization exercise was done on 10 children (five less than 23 months old and five 24–59 months old children) trainees who passed standardization exercise were allowed to start data collection. Anthropometric measuring instruments were calibrated every day before starting the daily activity. During data collection, regular supervision and monitoring of the overall activity were done by the supervisor and principal investigator.

### 2.11 Data processing and analysis

Data was reviewed for completeness and consistency before being cleaned, coded, and put into Epi data version 3.1 before being exported to SPSS software version 23 for analysis. The World Health Organization (WHO) 2006 Growth Reference Standard was used to compute and compare the WLZ. A kid with a WLZ less than 3 SD below the reference population was categorized as having severe wasting (2). To characterize the research population in respect to key factors, descriptive statistics were employed. Binary logistic regression analysis was used to examine the relationship between SAM and exposure factors. The Hosmer‐Lemeshow goodness‐of‐fit tests were used to assess the model's fitness. The Chi‐square and odds ratio (OR) with 95% confidence intervals were used to evaluate the connection between variables linked with the prevalence of child abuse. The factors associated with SAM in the bivariable model (*p* = 0.25) were then input and examined using a multivariable logistic regression model to determine the independent influence of distinct determinants on the occurrence of SAM. The level of statistical significance was set at *p* = 0.05.

### 2.12 Ethical consideration

Ethical clearance was obtained from Wallaga University's research and ethical review committee (RERC) (with protocol number **WU/RD/537/2014**). Permission was received from the East Wollega zone's zonal and Leqa Dulecha woreda health offices. Before the interview, informed verbal and written consents were sought from the children's parents/caretakers. After verbal agreement, illiterate moms were consented via their thumb prints. Mothers and caregivers of children with acute malnutrition were instructed on how to avoid and treat it, and for those instances that did not begin treatment, a link to therapeutic feeding clinics was established.

### 2.13 Terminology definition

1. **Cases**: Children with SAM whose MUAC ≤11.5 cm, WLZ <−3 SD or with bilateral pitting edema.
2. **Controls**: Children with normal nutritional status (without bilateral pitting edema or their MUAC >12.5 cm, their WLZ ≥−2 SD).
3. **Complementary feeding**: The process of starting to give additional foods and fluids to the child in addition to breast milk.
4. **Vaccinated**: A child who completes all EPI scheduled vaccine considered as fully vaccinated, a child who doesn't complete all EPI scheduled vaccine considered as not vaccinated.
5. **Exclusive breastfeeding**: Breastfeeding while giving no other food or liquid, not even water for infants up to the age of 6 months.
6. **Severe acute malnutrition**: A child whose weight for length is below −3 SD of the median WHO reference values.

## 3 RESULTS

### 3.1 Socio‐demographic characteristics

Among the total 256 study participants, 247 were included in the study with an overall response rate of 96.5%. The mean age of mothers or caregivers of cases and controls was 29.3 ± 5.25 and 27.75 + 5.33, respectively. Regarding educational status of mothers, 9 (15%) of cases and 28 (15%) of controls cannot read and write. Among the total study participants only 5 (8.3%) of cases and 22 (11.8) of controls were employed. Regarding the marital status of mothers/caregivers, 23 (38.3%) of cases and 45 (24.1%) of controls was living separated (Table 1).

**Table 1:** Socio‐demographic characteristics of study participants visiting health facilities in Leqa Dulacha district, East Wollega, Oromia, Ethiopia, 2022 (*n* = 247 children's mothers).

Variables	Categories	Controls (%)	Cases (%)	Total (%)
Maternal educational status	Cannot read $ write	28 (15.0)	9 (15.0)	37 (15.0)
	Read and write	24 (12.8)	8 (13.3)	32 (13.0)
	Primary level (1–8)	90 (48.1)	31 (51.7)	121 (49.0)
	Secondary level (9–12)	26 (13.9)	9 (15.0)	35 (14.2)
	College/university	19 (10.2)	3 (5.0)	22 (8.9)
Mather's occupation	Housewife	58 (31.0)	19 (31.7)	77 (31.2)
	Farmer	85 (45.5)	29 (48.3)	114 (46.2)
	Employed	22 (11.8)	5 (8.3)	27 (10.9)
	Others	22 (11.7)	7 (11.6)	29 (11.7)
Maternal marital status	Single/separated	45 (24.1)	23 (38.3)	68 (27.5)
	Paired	142 (75.9)	37 (61.7)	179 (72.5)
Father education	Illiterate	11 (5.9)	5 (8.3)	16 (6.5)
	Read and write	17 (9.1)	8 (13.3)	25 (10.1)
	Primary level (1–8)	67 (35.8)	28 (46.7)	95 (38.5)
	Secondary level (9–12)	56 (29.9)	10 (16.7)	66 (26.7)
	College/university	36 (19.3)	9 (15.0)	45 (18.2)
Father occupation	Farmer	100 (53.5)	33 (55.0)	133 (53.8)
	Merchant	37 (19.8)	14 (23.3)	51 (20.6)
	Employed	43 (23.0)	10 (16.7)	53 (21.5)
	Others	7 (3.7)	3 (5.0)	10 (4.0)
Ethnicity	Oromo	180 (96.3)	56 (93.3)	236 (95.5)
	Others	7 (3.7)	4 (6.7)	11 (4.5)
Religion	Orthodox	72 (38.5)	21 (35.0)	93 (37.7)
	Protestant	72 (38.5)	19 (31.7)	91 (36.8)
	Catholic	11 (5.9)	5 (8.3)	16 (6.5)
	Muslim	32 (17.1)	15 (25.0)	47 (19.0)

### 3.2 Child and family related characteristics

The children in the case and control groups had mean ages of 23.25 + 9.36 and 26.5 + 14.2 months, respectively. In contrast to the majority of controls, which were 106 (56.7%) male, the majority of children in cases 38 (63.3%) were female. Out of all the children in the family, a greater percentage of cases (47, or 78.3%) and controls (132, or 70.6%) were the second and older child in birth order. The total number of family members in 30 (50%) of the cases and 91 (48.7%) of the controls was greater than 5. As indicated by the table below (Table 2), several children under the age of five were present in the households of 34 (56.7%) of the cases and 78 (41.7%) of the controls (Table 2).

**Table 2:** Child and family related characteristics of study participants at health facilities in Leqa Dulacha district, East Wollega, Oromia, Ethiopia, 2022 (*n* = 247 children and mothers or caregiver pairs).

Variables	Categories	Control (%)	Cases (%)	Total (%)
Age of child	Less than 23 months	85 (45.5)	31 (51.7)	116 (47.0)
	Greater than 23 months	102 (54.5)	29 (48.3)	131 (53.0)
Sex of child	Female	81 (43.3)	38 (63.3)	119 (48.2)
	Male	106 (56.7)	22 (36.7)	128 (51.8)
Birth order	First child	55 (29.4)	13 (21.7)	68 (27.5)
	Second and above	132 (70.6)	47 (78.3)	179 (72.5)
Family size	Less than 4	96 (51.3)	30 (50.0)	126 (51.0)
	Greater than 4	91 (48.7)	30 (50.0)	121(49.0)
Birth interval	Less than 2 years	31 (16.6)	24 (40.0)	55 (22.3)
	Greater than 2 years	156 (83.4)	36 (60.0)	192 (77.7)
Number of under 5 children in the HH	One	109 (58.3)	26 (43.3)	135 (54.7)
	Two	78 (41.7)	34 (56.7)	112 (45.3)
Mather's autonomy	Low	97 (51.9)	32 (53.3)	129 (52.2)
	High	90 (48.1)	28 (46.7)	118 (7.8)

### 3.3 Environmental characteristics and health care utilization

Ten cases (16.7%) and 22 controls (11.8%) of the total study participants used unsafe water sources. Twelve (6.4%) of the study's total participants were controls, and 4(6.7%) of the cases were not utilizing latrines. Regarding the children's immunization status, 16 (26%) and 37 (19.8%) of the cases and controls, respectively, had not received the age‐appropriate vaccinations. Regarding vitamin, a supplementation, over half of the controls (79, 47.3%) and three‐quarters of the cases (41, 70.7%) did not receive it within the previous 6 months. In the previous 15 days, 30 cases (50%) and 40 controls (23.5%), respectively, became ill in one way or another. Ten (16.7%) of the cases and 12(6.4) of the controls indicated that there were family members with SAM (Table 3).

**Table 3:** Environmental characteristics and health care utilization of study participants in health facilities in Leqa Dulacha district, East Wollega, Oromia, Ethiopia, 2022 (*n* = 247 children and mothers or caregiver pairs).

Variables	Categories	Control (%)	Cases (%)	Total, *N* (%)
Source of drinking water	Protected	165 (88.2)	50 (83.3)	215 (87.0)
	Unprotected	22 (11.8)	10 (16.7)	32 (13.0)
Using Latrin	No	12 (6.4)	4 (6.7)	16 (6.5)
	Yes	175 (93.6)	56 (93.3)	231 (93.5)
Type of latrine	Household	121 (68.4)	36 (63.2)	157 (67.1)
	Communal	56 (31.6)	21 (36.8)	77 (32.9)
Immunization	Completed	82 (43.9)	27 (45.0)	109 (44.1)
	Up to date	68 (36.4)	17 (28.3)	85 (34.4)
	Defaulted	16 (8.6)	9 (15.0)	25 (10.1)
	Not started	21 (11.2)	7 (11.7)	28 (11.3)
Vitamin A	Yes	88 (52.7)	17 (29.3)	105 (46.7)
	No	79 (47.3)	41 (70.7)	120 (53.3)
De worming in the last 6 months	Yes	48 (27.9)	9 (16.4)	57 (25.1)
	No	54 (31.4)	17 (30.9)	71 (31.3)
	Not applicable	70 (40.7)	29 (52.7)	99 (43.6)
Illness in the last 15 days	Yes	44 (23.5)	30 (50.0)	74 (30.0)
	No	143 (76.5)	30 (50.0)	173 (70.0)
Type of illness	AFI	21 (47.7)	10 (33.3)	31 (41.9)
	RTI	18 (40.9)	5 (16.7)	23 (31.1)
	Diarrhea	5 (11.4)	15 (50.0)	20 (27.0)
Family member SAM	Yes	12 (6.4)	10 (16.7)	22 (8.9)
	No	175 (93.6)	50 (83.3)	225 (91.1)

### 3.4 Child feeding practice

When it came to breastfeeding, most cases (33, 55.0%) received only partial feedings, while most controls (122, 65.2%) received exclusive breastfeeding for the first 6 months of life. The majority of cases, 36 (87.8), and the control group, 94 (80.3), stopped nursing before they turned 24 months old. Ten (16.7%) of the study's total participants who were cases and 12 (6.4%) of the control group said they fed their child fewer than three times a day. In the 24 h before data collection, almost one‐third, 63 (33.7%), of controls and three‐quarters, 46 (76.7%), of cases experienced inadequate dietary diversity. Table 4 shows that among the 32 cases (53.3%) and 33 controls (17.6%) that were from food‐securing homes, more than half were cases (Table 4).

**Table 4:** Child feeding practice and food security of study participants visiting health facilities in Leqa Dulacha district, East Wollega, Oromia, Ethiopia, 2022 (*n* = 247 children and mothers or caregiver pairs).

Variables	Categories	Control (%)	Cases (%)	Total (%)
Type of breastfeeding before 6 m	Exclusive BF	122 (65.2)	27 (45.0)	149 (60.3)
	Partial BF	65 (34.8)	33 (55.0)	98 (39.7)
Age of starting complementary food	At 6 months	112 (59.9)	24 (40.0)	136 (55.1)
	Before 6 months	67 (35.8)	33 (55.0)	100 (40.5)
	Others	8 (4.3)	3 (5.0)	11 (4.5)
Current BF	No	117 (62.6)	40 (66.7)	157 (63.6)
	Yes	70 (37.4)	20 (33.3)	90 (36.4)
Frequency of BF	Less than 8 times per day	19 (27.1)	4 (19.0)	23 (25.3)
	Greater than 8 times per day	51 (72.9)	17 (81.0)	68 (74.7)
Weaning age	Less than 24 months	94 (80.3)	36 (87.8)	130 (82.3)
	Greater than 24 months	23 (19.7)	5 (12.2)	28 (17.7)
Bottle feeding	Yes	48 (25.7)	9 (15.0)	57 (23.1)
	No	139 (74.3)	51 (85.0)	190 (76.9)
Frequency of feeding per day	Less than 3 times per day	12 (6.4)	10 (16.7)	22 (8.9)
	Greater than 3 times per day	175 (93.6)	50 (83.3)	225 (91.1)
Training on Child feeding	No	164 (87.7)	55 (91.7)	219 (88.7)
	Yes	23 (12.3)	5 (8.3)	28 (11.3)
Source of information	No source	46 (24.6)	22 (36.7)	68 (27.5)
	Radio	95 (50.8)	25 (41.7)	120 (48.6)
	TV	31 (16.6)	10 (16.7)	41 (16.6)
	Health worker	15 (8.0)	3 (5.0)	18 (7.3)
Dietary diversity	Poor	63 (33.7)	46 (76.7)	109 (44.1)
	Good	124 (66.3)	14 (23.3)	138 (55.9)
Food security (HFIAS)	Food secured	154 (82.4)	28 (46.7)	182 (73.7)
	Food insecure	33 (17.6)	32 (53.3)	65 (26.3)

### 3.5 Parents' behavioral factors

**Figure 3:**
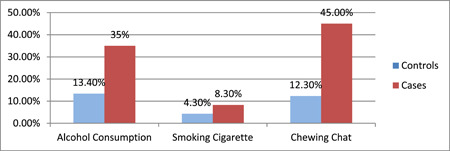
Behavioral factors of study participants visiting health facilities in Leqa Dulacha district, East Wollega, Oromia, Ethiopia, 2022 (*n* = 247 children and mothers or caregiver pairs).

Children's parents' experience of substance use, such as Alcohol consumption, smoking cigarette and chewing chat was assessed. The most commonly used substance was chat (45% of cases and 12.3% of controls). The result has been displayed by the below figure (Figure 3).

### 3.6 Determinants of SAM

Candidates for multivariable logistic regression analysis were variables that demonstrated relationships with a *p* < 0.05. The multicollinearity test was passed by every candidate variable (all candidate variable correlation coefficients at *p* < 0.05). The resultant model has a satisfactory fit, according to the Hosmer‐Lemeshow goodness of fit test (*p* > 0.05 and *χ* ^2^ = 5.723). Ultimately, the variables that had a *p*‐value of less than 0.05 in multivariable logistic regression analysis were identified as the SAM determinants.

According to multivariable logistic modeling, children with SAM were approximately 2.8 times more likely than their contemporaries to not have received vitamin A during the 6 months' prior [adjusted odds ratio (AOR) = 2.89, 95% confidence interval (CI): 1.14–7.30]. Children who had been ill within the 15 days before the survey had a risk of SAM that was more than four times higher than that of children who had not been ill over the previous 15 days [AOR = 4.12, 95% CI: 1.68–10.07]. Three times as many children as those who ate foods with poor dietary diversity had the potential to acquire SAM [AOR = 3.04, 95% CI: 1.18–7.78] (Table 5).

**Table 5:** Multivariable logistic regression analysis of factors associated with Severe Acute Malnutrition among 6–59 months old children visiting health facilities in Leqa Dulacha district, East Wollega, Oromia, Ethiopia, 2022 (*n* = 247 children and mothers or caregiver pairs).

Variables	Cases, *N* (%)	Control, *N* (%)	COR (95% CI)	AOR (95% CI)
**Marital status**				
Single/separated	23 (38.3)	45 (24.1)	1.96 (1.06, 3.64)	2.420 (0.96, 6.09)
Paired	37 (61.7)	142 (75.9)	1	1
**Sex of child**				
Female	38 (63.3)	81 (43.3)	2.26 (1.24, 4.11)	2.197 (0.94, 5.12)
Male	22 (36.7)	106 (56.7)	1	1
**Birth interval**				
Less than 2 years	24 (40.0)	31 (16.6)	3.35 (1.76, 6.39)	1.681 (0.62, 4.56)
Two years or more	36 (60.0)	156 (83.4)	1	1
**Number of** <**5 children**				
Two	34 (56.7)	78 (41.7)	1.83 (1.01, 3.28)	1.02 (0.42, 2.44)
One	26 (43.3)	109 (58.3)	1	1
**Vitamin A supplied in the last 6 months**				
No	41 (70.7)	79 (47.3)	2.67 (1.41–5.10)	2.89 (1.14, 7.30)^a^
Yes	17 (29.3)	88 (52.7)	1	1
**Illness in the last 15 days**				
Yes	30 (50.0)	44 (23.5)	3.25 (1.77, 5.97)	4.12 (1.69, 10.0)^a^
No	30 (50.0)	143 (76.5)	1	1
**Family SAM**				
Yes	10 (16.7)	12 (6.4)	2.92 (1.19–7.14)	1.08 (0.25, 4.70)
No	50 (83.3)	175 (93.6)	1	1
**Brest feeding before 6m**				
Partial BF	33 (55.0)	65 (34.8)	2.29 (1.27, 4.14)	1.29 (0.49, 3.39)
Exclusive BF	27 (45.0)	122 (65.2)	1	1
**Number of feeding/day**				
Less than 3 times/day	10 (16.7)	12 (6.4)	2.92 (1.19–7.15)	1.28 (0.33, 4.90)
Three or more times/day	50 (83.3)	175 (93.6)	1	1
**DDS**				
Poor	46 (76.7)	63 (33.7)	6.47 (3.30, 12.64)	3.04 (1.19, 7.79)^a^
Good	14 (23.3)	124 (66.3)	1	1
**HFIAS**				
Food Insecure	32 (53.3)	33 (17.6)	5.33 (2.84, 10.03)	4.02 (1.54, 10.49)^a^
Food Secured	28 (46.7)	154 (82.4)	1	1
**Drinking alcohol**				
Yes	21 (35.0)	25 (13.4)	3.49 (1.77, 6.87)	3.14 (1.10, 8.94)^a^
No	39 (65.0)	162 (86.6)	1	1
**Chewing chat**				
Yes	27 (45.0)	23 (12.3)	5.83 (2.10, 11.40)	3.48 (1.33, 9.13)^a^
No	33 (55.0)	164 (87.7)	1	1

Abbreviations: AOR, adjusted odds ratio, CI, confidence interval; COR, crude odds ratio.

^a^ Statistically significant at *p* < 0.05, 1 = reference category.

Compared to children from households where food was secure, children from households with food insecurity were more than four times more likely to have SAM [AOR = 4.02, 95% CI: 1.54–10.49]. Moreover, children of alcoholic parents had a 3.14‐fold increased risk of developing SAM [AOR = 3.14, 95% CI: 1.10–8.94] compared to offspring of nonalcoholic parents. In a similar vein, children were 3.48 times more likely than their contemporaries to have SAM if their parents were chewing chat [AOR = 3.48, 95% CI: 1.32–9.13] (Table 5).

## 4 DISCUSSION

The results of this investigation showed a strong correlation between SAM in children and the absence of vitamin A supplementation during the 6 months before to data collection. Children who had not received vitamin A within the 6 months before the data collection were at a higher risk of developing SAM compared to those who had received vitamin A within the designated time frame. This data is consistent with research findings from Bangladesh^19^ and Gahana^18^ which reported that there was significant association between vitamin A supplementation status of children and SAM among children. This could be because vitamin A is known to be one of the direct causes of SAM and is involved in the immune system's development as well as regulating cellular immunological responses that shield children from illness.^20^ Children who had not received vitamin A may therefore have missed this chance, making it easier for them to acquire SAM.

The current study also demonstrated that among children aged 6–59 months, sickness in the 2 weeks preceding to data collection was another independent contributing factor to SAM. Compared to their peers, children who had experienced any sickness (such as fever, cough, or diarrhea) 2 weeks before the data collection were more likely to have had SAM. This outcome is consistent with the findings of other research projects carried out globally.^9^, ^14^, ^16^ This could be explained by the fact that children's illnesses cause malabsorption, decreased appetite, and decreased calorie intake. On the other side, a disease causes the body to use more energy because of an accelerated metabolism. In addition, the children's elevated body temperature from inflammations will burn down the stored calories.^21^ As a result, children who got illness may face these risks more than those who did not get and developed SAM.

The current study found a substantial correlation between children's SAM and dietary variety score. The odds of SAM were higher for kids who ate food with inadequate dietary diversity within the 24 h before the survey than for kids who ate food with good dietary diversity during that same period. This outcome matched the findings of a research conducted in Chad^11^ which revealed that children's status of dietary diversity was significantly associated with their nutritional status. This might be due to the fact that, children who feed on diversified diets can get adequate variety of nutrients and sufficient energy needed as recommended daily allowance for their age. On the other hand, parents who feed their children from poor dietary diversified foods may expose the children to imbalanced diet and low energy intake for which SAM can be occurred.

Another component in this study that was substantially linked with SAM was the food security level of the household. Compared to their peers, children from homes experiencing food hardship had a higher risk of developing SAM. This conclusion is consistent with a case control study conducted in Nepal that found children from food‐insecure households had a higher risk of developing SAM compared to children from food‐secure households (35). In a similar vein, research from Northwest Ethiopia (41) confirms this outcome. This could be because there isn't enough food available or because households don't have the money to buy enough food; kids might be fed less food than is appropriate for their age. Furthermore, families with inadequate food supplies may give their kids low‐quality meals, which could harm the kids.

In this study parents drinking alcohol was found to be significantly associated with SAM among children. Children those whose parents were drinking alcohol were more likely to have SAM when compared with that of those whose parents were not drinking. The result is not supported by the report of study conducted in India^22^ in which the association between drinking alcohol and SAM was insignificant. This is probably due to the fact that parents may need and spend more budgets to purchase alcoholic drinks rather than purchasing food for their children. It may also divert the parents' attention from children to seek for alcoholic drinks.

This study also identified that Parent's Chewing chat was significantly associated with SAM among children. Children who were from parents who chewed chat were more likely to develop SAM when compared to those whose parents were not chewing. The reason could be due to the fact that parents who were chewing chat may spend more money to buy chat which exposes the household for food insecurity. In addition to this, chewing chat may also take the child caring time of parents and interferes the parents and child bond.

## 5 LIMITATIONS

As data was collected by the health workers the result may be subjected to social desirability bias. It was based on respondent's self‐reported data and tracking of exposure status retrospectively, which could be prone to recall bias.

## 6 CONCLUSION

In this study, Children's status of Vit‐A supplementation, Child feeding practice such as dietary diversity, household food security status, Illness in the last 15 days, parents' substance use, such as drinking alcohol and Chewing chat were identified to be independent determinants of SAM among 6–59 months old children.

To address these determinants and improve the nutritional status of children, Health extension and health workers should give education for the community on early detecting the sick child, the importance of dietary diversification and encourage parents to provide their children with as much as possible more diversified food and parents should change their life style.

In addition, exclusive breastfeeding is economically preferable to other methods, supports policies to reduce the time cost of exclusive breastfeeding (e.g., paid maternity leave and maternal cash transfers), and addresses the importance of mother's mental health to ensure successful breastfeeding.

## AUTHOR CONTRIBUTIONS

**Garoma Begna**: Conceptualization; Investigation; Writing—original draft; Methodology; Funding acquisition; Validation; Visualization; Writing—review & editing; Software; Formal analysis; Project administration; Data curation; Supervision; Resources. **Haile Bikila**: Conceptualization; Methodology; Software; Data curation; Supervision; Formal analysis; Project administration; Visualization; Validation; Investigation; Funding acquisition; Resources; Writing—review & editing; Writing—original draft. **Bayise Biru**: Conceptualization; Methodology; Software; Data curation; Supervision; Formal analysis; Validation; Investigation; Funding acquisition; Visualization; Project administration; Resources; Writing—review & editing; Writing—original draft. **Debelo Diriba**: Conceptualization; Investigation; Funding acquisition; Writing—original draft; Writing—review & editing; Visualization; Validation; Methodology; Formal analysis; Software; Project administration; Data curation; Supervision; Resources. **Chimdesa Tolera**: Data curation; Supervision; Resources; Project administration; Formal analysis; Software; Methodology; Validation; Visualization; Writing—review & editing; Writing—original draft; Funding acquisition; Investigation; Conceptualization. **Ra'el Dessalegn**: Conceptualization; Investigation; Funding acquisition; Writing—original draft; Writing—review & editing; Visualization; Validation; Methodology; Software; Formal analysis; Project administration; Resources; Supervision; Data curation. **Temesgen Tafesse**: Conceptualization; Investigation; Funding acquisition; Writing—original draft; Visualization; Validation; Methodology; Writing—review & editing; Project administration; Formal analysis; Software; Supervision; Resources; Data curation. **Dessalegn Amenu**: Conceptualization; Investigation; Funding acquisition; Writing—review & editing; Visualization; Validation; Methodology; Writing—original draft; Software; Formal analysis; Project administration; Data curation; Supervision.

## CONFLICT OF INTEREST STATEMENT

The authors declare no conflicts of interest.

## TRANSPARENCY STATEMENT

The lead author Dessalegn Amenu affirms that this manuscript is an honest, accurate, and transparent account of the study being reported; that no important aspects of the study have been omitted; and that any discrepancies from the study as planned (and, if relevant, registered) have been explained.

## Data Availability

Data sharing not applicable to this article as no datasets were generated or analysed during the current study.
